# A New Volume

**Published:** 1857-05

**Authors:** 


*A New Volume.*—With the present number we leave the monysyllabic numbers and pass into our "teens." With the June number will commence the Thirteenth Volume of the Journal. During twelve years it has been regularly and unfailingly issued, and in all that time there has been but a single change in its editorial control. At no previous time has it boasted so large and sound a subscription list, and we may add, with pardonable vanity, that at no time has it better deserved success. We have made arrangements for the forthcoming volume which cannot fail to improve it. We have a list of collaborators equal in talent, if not in numbers, to any other in the country. Profs. Flint, Hamilton, Rochester, White and others are reliable for frequent contributions, while among the ranks of the profession we have many other names who have promised us their assistance. One new feature will be the regular publication of clinical reports by Dr. Austin W. Nichols, of cases observed at the Buffalo Hospital of the Sisters of Charity, which will be especially valuable for their practical character. During the coming year we expect to witness the inauguration of another hospital in Buffalo, which will furnish a large amount of clinical instruction and which we anticipate will be made tributary to the Journal. The Proceedings of the Buffalo Medical Association — always interesting and valuable — will be regularly published in each number. We are fully awake to the fact that no single man can make a good medical monthly, and that the elaborate thoughts of occasional contributors who write only because they have something to say, are always to be preferred to hasty editorial scribblings. Bearing this in mind we have withdrawn somewhat from the use of our personal pen in favor of others more thoroughly qualified.

As to our eclectic department we recognize its value, but with what we know to be the sentiments of our readers, we prefer original matter. An examination of the past volume will show that no other medical monthly in the country has published so large a proportion of original articles as our own.

With renewed energy and with brighter prospects for the future than we had anticipated, we enter upon our new volume with a determination that our increasing experience shall add to its interest and make it worthy of the warm support it has received, not only from the profession of the West, but of the East, the South, and Canada.

